# Research on Energy Efficiency of NOMA–SWIPT Cooperative Relay Network Using GS-*DinkelBach* Algorithm

**DOI:** 10.3390/s21175720

**Published:** 2021-08-25

**Authors:** Ninghao Zhou, Jinfeng Hu, Jia Hou

**Affiliations:** 1 School of Electronics & Information, Soochow University, Suzhou 215000, China; Chaos.NinghaoZhou@gmail.com; 2 Yangtze Delta Region Institute (Quzhou), University of Electronic Science and Technology of China, Quzhou 324000, China; hujf@uestc.edu.cn

**Keywords:** simultaneous wireless information and power transfer (SWIPT), non-orthogonal multiple access (NOMA), cooperative network, energy efficiency (EE)

## Abstract

In order to improve the energy efficiency (EE) performance of cooperative networks, this study combines non-orthogonal multiple access (NOMA) with simultaneous wireless information and power transfer (SWIPT) technologies to construct a cooperative relay network composed of one base station (BS), multiple near users, and one far user. Based on the network characteristics, a time-division resource allocation rule is proposed, and EE formulas regarding direct-link mode and cooperative mode are derived. Considering user selection and decoding performance, to obtain the optimal EE, this study utilizes a *DinkelBach* iterative algorithm based on the golden section (GS-*DinkelBach*) to solve the EE optimization problem, which is affected by power transmitted from the BS, achievable rates under three communication links, and quality of service (QoS) constraints of users. The simulation results show that the GS-*DinkelBach* algorithm can obtain precise EE gains with low computational complexity. Compared with the traditional NOMA–SWIPT direct-link network model and the relay network model, the optimal EE of the established network model could be increased by 0.54 dB and 1.66 dB, respectively.

## 1. Introduction

According to the Cisco annual Internet report (2018–2023) white paper, the share of machine-to-machine (M2M) connections will grow from 33 percent in 2018 to 50 percent by 2023, the fifth generation (5G) speeds will be 13 times higher than the average mobile connection, and the average 5G connection speed will reach 575 Mbps by 2023 [1]. The rapid growth in the number of communication devices and transmission rates will cause an explosion of data traffic. To satisfy the requirement of communication traffic in 5G, resource allocation rules and corresponding technologies of communication networks should be improved, and energy consumption should be taken into consideration [2].

As a promising candidate for 5G, non-orthogonal multiple access (NOMA) puts forward the concept of a power domain that is different from the time domain, frequency domain, and space domain. Compared with traditional orthogonal multiple access (OMA) techniques, NOMA allows each sub-carrier to simultaneously serve multiple terminals so that significant spectral efficiency (SE) enhancement can be obtained [3,4]. Users sharing the same spectrum will cause great mutual interference, and the solution to this situation is the utilization of successive interference cancellation (SIC) technology at the receiver so that the demanded information can be correctly decoded. Apart from SIC, other technologies, such as multiple-input multiple-output (MIMO) [5], wireless power communication (WPC) [6], and sparse code multiple access (SCMA) [7,8], used in NOMA have also been researched in recent years, with a predominant focus on the optimization of codec and resource allocation.

As another form of technology considering the power domain, simultaneous wireless information and power transfer (SWIPT) has also attracted attention due to its potential in solving the problem of excessive energy consumption of equipment. SWIPT refers to the technology that allows for information and energy from a radio frequency signal to be simultaneously received by using the radio frequency signal’s ability to not only transmit information but also carry energy. The concept of SWIPT was firstly proposed by L. R. Varshney in 2008, and it was explored in relation to the performance tradeoff between energy and information rates [9]. Two kinds of common transmission methods have been developed to combine and distinguish information signals and power signals, i.e., the time-switching (TS) method and the power-splitting (PS) method. Information signals and power signals are transmitted in a time-division-multiplexing manner with the TS method or superimposed with a certain power ratio with the PS method [10]. To match the transmission methods, the receiver architecture should be equipped with the integration of an information decoder (ID) and energy harvester (EH).

The relaying method is described as an efficient means to improve the quality of long-distance communication. In order to fix the problem of far users receiving weak signals, relays closer to the signal source are utilized to forward signals and improve the received signal strength. Through node positions, diversity methods, and related protocols, resources can be efficiently exploited rather than wasted [11].

In recent years, the combination of NOMA and SWIPT technologies in cooperative networks has received a considerable amount of attention. For example, in [12,13,14], different types of NOMA–SWIPT cooperative relay network models are proposed, and the resource allocation optimization methods is studied with the goal of outage performance of the networks. On this basis, other studies also comprehensively consider the impact of the throughput [15,16,17,18,19], diversity gain [20,21,22], and user transmission distance [23,24], verifying that the cooperative relay network of NOMA–SWIPT fusion has a good prospect in energy and resource optimization. Due to the adoption of the relay cooperation scheme, under the multi-relay model, the reasonable formulation of the relay selection methods can effectively improve the network performance. A novel buffer-aided relay selection scheme is proposed in [25], and the corresponding performance in different communication links and modes is analyzed. In [26], a single-source, randomly located multi-relay network is examined to compare the communication performance in direct-link and cooperative relay. The system model is expanded to multiple sources and users in [27]. The research conducted in [28] also considers the bit error rate (BER) performance of multiple users after the *m*th best relay selection from a practical perspective.

The NOMA–SWIPT cooperative relay network protocol and architecture based on half duplex (HD) and full duplex (FD) relays are also respectively proposed in [29,30]. The problem of energy harvesting and information forwarding of relay nodes is examined to identify the solution to joint optimization of resource allocation in network nodes. Furthermore, in [31], a hybrid HD/FD relay selection is proposed to make the network more efficient and stable. The abovementioned works further prove that the cooperative relay network architecture based on NOMA–SWIPT technology can not only obtain the gain of transmission performance but also optimize energy consumption during the transmission of the system.

However, most of the abovementioned research focuses on analyzing network outage performance or transmission performance under the premise of using SWIPT, but there is a lack of analysis and optimization of the energy efficiency (EE) of networks. Therefore, inspired by current research, this paper takes a NOMA–SWIPT cooperative relay network consisting of one base station, multiple near users, and one far user into consideration. In order to satisfy resource scheduling under different network conditions, the system is divided into two modes, i.e., direct-link mode and cooperative mode, and a time division resource allocation rule is proposed. Through the analysis of channel state information (CSI) and performance parameters of nodes in the network, an optimal near user is selected. Then, the network EE performance is studied on the condition of ensuring the far user’s and optimal near user’s information reception. The EE formulas constrained by the power transmitted from the BS, achievable rates under different communication channels and the quality of service (QoS) of users, are given from the perspective of direct-link mode and cooperative mode, respectively, under the network architecture proposed. Through derivation, it can be concluded that the EE formulas in different modes of the network are concave functions, so the corresponding EE optimization problems have optimal solutions. On this basis, a *DinkelBach* iterative algorithm based on golden section (GS-*DinkelBach*) is proposed. System simulation results show that the algorithm can obtain precise EE gains with good convergence and lower computational complexity. In addition, decoding and EE performance of users in different modes are presented. Compared with the traditional NOMA–SWIPT non-cooperative network model and non-direct-link network model, the optimal energy efficiency of the proposed network model can be improved by 0.54 dB and 1.66 dB, respectively.

The remainder of the paper is structured as follows: In Section 2, the system model is constructed. Section 3 presents the corresponding resource allocation rule. In Section 4, EE optimization problems are derived, and the GS-*DinkelBach* algorithm is proposed in Section 5. Simulation and numerical results are given and analyzed in Section 6, which is followed by a conclusion in Section 7.

## 2. System Model

**Figure 1:**
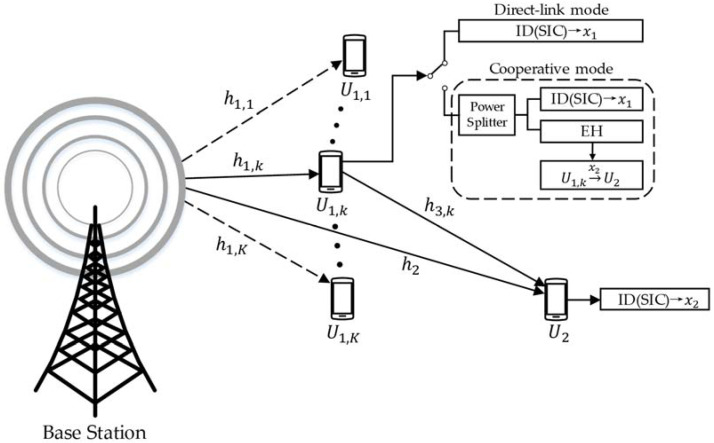
Downlink NOMA–SWIPT cooperative relay network.

As shown in Figure 1, the considered downlink cooperative NOMA network based on SWIPT includes one BS, K near users $U_{1,k}$ (k=1,…,K), and one far user $U_{2}$.

In the constructed system model, $h_{1,k}$, $h_{2}$, and $h_{3,k}$ are the channel fading coefficients of the $BS-U_{1,k}$ link, the $BS-U_{2}$ link, and the $U_{1,k}-U_{2}$ link, respectively. $U_{1,k}$ and $U_{2}$ are both equipped with single antennae and use successive interference cancellation (SIC) technology to decode the demanded signals $x_{1}$ and $x_{2}$. The BS functions as a signal-transmitting source. Since $U_{1,k}$ is closer to the BS, it could perform as an energy harvester and a cooperative relay. On the other hand, as the far user, the signal receiving quality of $U_{2}$ is worse. Thus, two modes of network situations are shown in Figure 1, i.e., direct-link mode and cooperative mode, to ensure the communication quality of the network.

The NOMA = modulated signal transmitted by the BS can be expressed as

(1) $$x=\sqrt{P_{1}}x_{1}+\sqrt{P_{2}}x_{2},$$

where $x_{1}$ and $x_{2}$ are the signals required by $U_{1,k}$ and $U_{2}$ with power $E\left[{\left|x_{1}\right|}^{2}\right]=E\left[{\left|x_{2}\right|}^{2}\right]=1$, and $P_{1}$ and $P_{2}$ are the power allocated for $x_{1}$ and $x_{2}$ using NOMA technology. $P_{1}=aP_{s}$, $P_{2}=\left(1-a\right)P_{s}$, where $P_{s}$ is the BS transmit power and a is the BS power allocation factor. In the proposed network model, the channel power gain of the $BS-U_{2}$ link is less than that of the $BS-U_{1,k}$ link, so the BS needs to allocate more power for $U_{2}$, i.e., $0<a<\frac{1}{2}$.

In order to distinguish between near users and far users, this study assumes a distance-based Rayleigh fading channel scenario and considers the distance-dependent function utilized in [26] as deterministic influence for path loss, which is expressed as

(2) $$\theta =\varsigma {\left(1+d\right)}^{-\varrho }$$

where ς and ϱ are the propagation coefficients and d denotes the distance of two communication nodes. The function θ should satisfy $P_{R}=\theta {\left|h\right|}^{2}P_{T}$, where $P_{T}$ and $P_{R}$ are transmitted power and received power, respectively, and ${\left|h\right|}^{2}$ is the Rayleigh channel gain.

Thus, in direct-link mode, the received signals of two users are given by

(3) $$y_{1,k}^{direct}=h_{1,k}\sqrt{\theta_{1,k}}x+n_{1,k},$$

(4) $$y_{2}^{direct}=h_{2}\sqrt{\theta_{2}}x+n_{2}$$

where $n_{1,k}$ and $n_{2}$ are the additive white Gaussian noise (AWGN) in the corresponding communication link, $n_{1,k}\sim CN\left(0,\sigma_{1,k}^{2}\right),\;n_{2}\sim CN\left(0,\sigma_{2}^{2}\right)$.

In cooperative mode, $U_{1,k}$ switches into a half-duplex SWIPT relay. The receiving antenna of $U_{1,k}$ is followed by a power splitter. The PS factor of $U_{1,k}$ is ρ, 0<ρ<1. According to the system model proposed in this study, the $\left(1-\rho \right)$ proportion of the total signal power received from the BS in $U_{1,k}$ is utilized to decode information, and the rest, i.e., ρ proportion, is harvested as power for forwarding $x_{2}$ to $U_{2}$. Thus, the energy harvested in $U_{1,k}$ is

(5) $$P_{r,k}=\eta \rho \theta_{1,k}{\left|h_{1,k}\right|}^{2}P_{s},$$

where η is the energy conversion efficiency of $U_{1,k}$, 0<η<1. The signal used for user detection is expressed as

(6) $$y_{1,k}^{coop}=\sqrt{1-\rho }y_{1,k}^{direct}.$$

Here, we ignore the additional loss of $U_{1,k}$ in the energy transforming process’ i.e., the energy harvested by $U_{1,k}$ is completely converted into the transmission power for $U_{1,k}-U_{2}$. Thus, the received signal of $U_{2}$ from $U_{1}$ is given as

(7) $$y_{2}^{relay}=h_{3,k}\sqrt{\theta_{3,k}P_{r,k}}x_{2}+n_{3,k},$$

where $n_{3,k}$ is the AWGN under the $U_{1,k}-U_{2}$ link,$\;n_{3,k}\sim CN\left(0,\sigma_{3,k}^{2}\right)$.

Therefore, in cooperative mode, considering maximal ratio combining (MRC), the total signal $U_{2}$ received in time slot T is given as

(8) $$y_{2}^{coop}=h_{2}\sqrt{\theta_{2}P_{1}}x_{1}+\left(h_{2}\sqrt{\theta_{2}P_{2}}+h_{3,k}\sqrt{\theta_{3,k}P_{r,k}}\right)x_{2}+n_{2}+n_{3,k}.$$

## 3. Resource Allocation Rule

**Figure 2:**
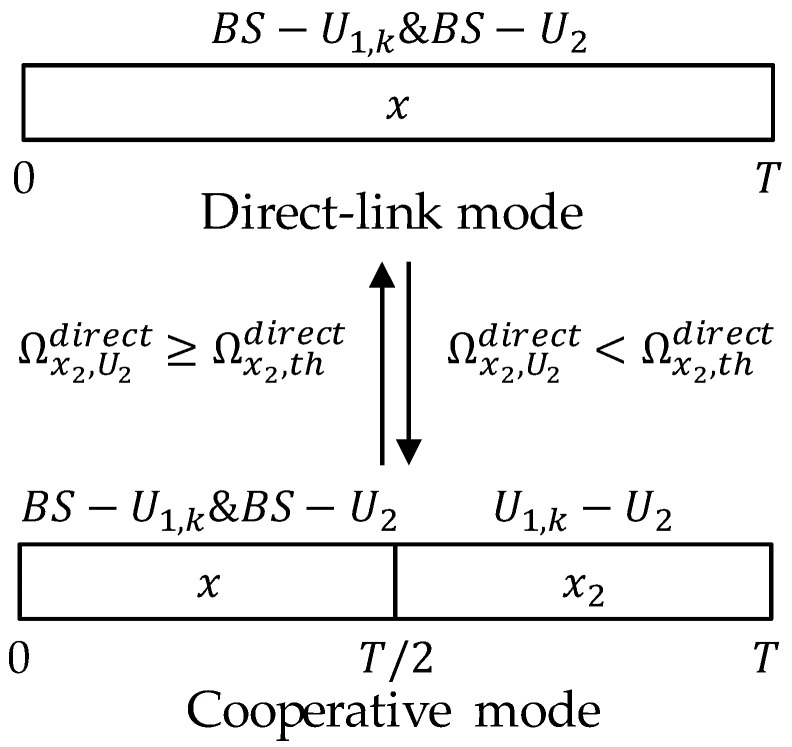
Time division resource allocation rule.

As mentioned in Section 2, in order to ensure the quality, this paper assumes that the NOMA–SWIPT cooperative relay network model proposed in Figure 1 can achieve mode switching in a working time slot T according to proposed time-division resource allocation rule. The rule composed of two modes is shown in Figure 2.

The mode switching of the proposed network depends on the CSI and achievable rates in three communication links. Let the target rates for $U_{1,k}$ and $U_{2}$ to correctly decode $x_{1}$ and $x_{2}$ be $R_{1}$ and $R_{2}$, respectively. Thus, the SINR thresholds in different modes should be expressed as $\Omega_{x_{1},th}^{direct}=2^{R_{1}}-1$, $\Omega_{x_{2},th}^{direct}=2^{R_{2}}-1$, $\Omega_{x_{1},th}^{coop}=2^{2R_{1}}-1$, $\Omega_{x_{2},th}^{coop}=2^{2R_{2}}-1$.

At the beginning of each working time slot T, the system will compare the CSI condition of the $BS-U_{2}$ link and transmission SINR for $U_{2}$ to decode $x_{2}$. If the signal to interference plus noise ratio (SINR) of $U_{2}$ is greater than threshold, i.e., $\Omega_{x_{2},U_{2}}^{direct}\geq \Omega_{x_{2},th}^{direct}$, $U_{2}$ will send a 1 bit ACK to the BS and $U_{1,k}$; then, the network will stay in direct-link mode, where

(9) $$\Omega_{x_{2},U_{2}}^{direct}=\frac{\theta_{2}{\left|h_{2}\right|}^{2}P_{2}}{\theta_{2}{\left|h_{2}\right|}^{2}P_{1}+\sigma_{2}^{2}}.$$

In this mode, $U_{1,k}$ and $U_{2}$ will directly receive the radio frequency (RF) signal sent by the BS only.

On the other hand, if the SINR of $U_{2}$ is lower than the threshold, i.e., $\Omega_{x_{2},U_{2,k}}^{direct}<\Omega_{x_{2},th}^{direct}$, $U_{2}$ will send a 1 bit NACK to the BS and $U_{1,k}$; then, the network will switch into cooperative mode. In this mode, the working time slot is divided into two halves with a length of T/2. In the first time slot, $U_{1,k}$ receives a superimposed signal and functions as an RF signal energy harvester. In the second time slot, $U_{1,k}$ functions as a cooperative relay, which could forward the cooperation signal to $U_{2}$ using RF energy harvested in the first time slot.

According to the system model and time division resource allocation rule summarized, achievable rates in direct-link mode and cooperative mode could be derived under CSI constraints.

In direct-link mode, $U_{1,k}$ first considers $x_{1}$ as interference to decode the information of $x_{2}$ through SIC technology. Thus, the achievable rate for $U_{1,k}$ to decode $x_{2}$ under the $BS-U_{1,k}$ link in direct-link mode is given by

(10) $$R_{x_{2},U_{1,k}}^{direct}=\log_{2}\left(1+\Omega_{x_{2},U_{1,k}}^{direct}\right),$$

where

(11) $$\Omega_{x_{2},U_{1,k}}^{direct}=\frac{\theta_{1,k}{\left|h_{1,k}\right|}^{2}P_{2}}{\theta_{1,k}{\left|h_{1,k}\right|}^{2}P_{1}+\sigma_{1,k}^{2}}.$$

Then, the information of $x_{2}$ will be discarded to obtain the demanded information of $U_{1,k}$, i.e., $x_{1}$. The achievable rate is given by

(12) $$R_{x_{1},U_{1,k}}^{direct}=\log_{2}\left(1+\Omega_{x_{1},U_{1,k}}^{direct}\right),$$

where

(13) $$\Omega_{x_{1},U_{1,k}}^{direct}=\frac{\theta_{1,k}{\left|h_{1,k}\right|}^{2}P_{1}}{\sigma_{1,k}^{2}}.$$

As a far user, $U_{2}$ only needs to decode the information of $x_{2}$ through the same technology in $U_{1,k}$, and the achievable rate is given by

(14) $$R_{x_{2},U_{2}}^{direct}=\log_{2}\left(1+\Omega_{x_{2},U_{2}}^{direct}\right),$$

In cooperative mode, the third communication link, i.e., the $U_{1,k}-U_{2}$ link, is built. $U_{1,k}$ will re-encode $x_{2}$ instead of discarding it. Different from direct-link mode, the power splitter in $U_{1,k}$ will be utilized through on-off keying. Thus, the PS factor is considered in the formula. The achievable rates for $U_{1,k}$ to decode $x_{1}$ and $x_{2}$ under the $BS-U_{1,k}$ link in cooperative mode are respectively given by

(15) $$R_{x_{1},U_{1,k}}^{coop}=\log_{2}\left(1+\Omega_{x_{1},U_{1,k}}^{coop}\right),$$

(16) $$R_{x_{2},U_{1,k}}^{coop}=\log_{2}\left(1+\Omega_{x_{2},U_{1,k}}^{coop}\right),$$

where

(17) $$\Omega_{x_{1},U_{1,k}}^{coop}=\frac{\left(1-\rho \right)\theta_{1,k}{\left|h_{1,k}\right|}^{2}P_{1}}{\sigma_{1,k}^{2}},$$

(18) $$\Omega_{x_{2},U_{1,k}}^{coop}=\frac{\left(1-\rho \right)\theta_{1,k}{\left|h_{1,k}\right|}^{2}P_{2}}{\left(1-\rho \right)\theta_{1,k}{\left|h_{1,k}\right|}^{2}P_{1}+\sigma_{1,k}^{2}}.$$

The change in $U_{1,k}$’s function will not affect the achievable rates for $U_{2}$ to decode $x_{2}$ under the $BS-U_{2}$ link in the first time slot. Moreover, $U_{2}$ also receives the re-encoded signal forwarded from $U_{1,k}$ in the second time slot. Thus, as a receiver using MRC technology, the achievable rate for $U_{2}$ to decode $x_{2}$ is given as

(19) $$R_{x_{2},U_{2}}^{coop}=\log_{2}\left(1+\Omega_{x_{2},U_{2}}^{coop}\right),$$

where

(20) $$\Omega_{x_{2},U_{2}}^{coop}=\frac{\theta_{2}{\left|h_{2}\right|}^{2}P_{1}}{\theta_{2}{\left|h_{2}\right|}^{2}P_{1}+\sigma_{2}^{2}}+\frac{\theta_{3,k}{\left|h_{3,k}\right|}^{2}P_{r}}{\sigma_{3,k}^{2}}.$$

It should be noted that due to the SINR of $U_{1,k}$ and $U_{2}$ in different modes, the comparison of $\Omega_{x_{1},U_{1,k}}^{direct}$ and $\Omega_{x_{1},th}^{direct}$, $\Omega_{x_{2},U_{2}}^{direct}$ and $\Omega_{x_{2},th}^{direct}$, $\Omega_{x_{1},U_{1,k}}^{coop}$ and $\Omega_{x_{1},th}^{coop}$, and $\Omega_{x_{2},U_{2}}^{coop}$ and $\Omega_{x_{2},th}^{coop}$ will influence whether the network operates successfully.

To obtain optimal system performance, a feasible $U_{1,k}$ should be selected. In direct-link mode, the standard to achieve near-user selection is that the channel gain of the $BS-U_{1,k}$ link should be optimal compared with others. Compared with direct-link mode, an extra link in cooperative mode should be considered. Based on the partial relay selection (PRS) scheme [32], we prioritize the optimal channel gain of the $BS-U_{1,k}$ link so that $U_{1,k}$’s power for forwarding signals and communication quality with the BS can be ensured, which is the premise of cooperative communication. Thus, the standard for optimal near user selection can be expressed as

(21) $$\underset{i=1,\ldots ,K}{\max}\;\theta_{1,i}{\left|h_{1,i}\right|}^{2}.$$

## 4. Energy Efficiency Analysis

As the ratio of data transmission to energy consumption, EE is regarded as a comprehensive standard to represent the network performance and considered as the objective function in optimization problems for wireless networks [33]. In this section, EE formulas and corresponding optimization problems in different network modes are derived.

### 4.1. EE Analysis in Direct-Link Mode

In direct-link mode, the network only operates the $BS-U_{1,k}$ and $BS-U_{2}$ links. According to the definition of EE and the correlation formulas, the analytical expression of EE in direct-link mode is given as

(22) $$EE_{k}^{direct}=\frac{T\left(R_{x_{1},U_{1,k}}^{direct}+R_{x_{2},U_{2}}^{direct}\right)}{\left(P_{s}+P_{c}\right)T}\;=\frac{\log_{2}\left(1+\frac{\theta_{1,k}{\left|h_{1,k}\right|}^{2}P_{1}}{\sigma_{1,k}^{2}}\right)+\log_{2}\left(1+\frac{\theta_{2}{\left|h_{2}\right|}^{2}P_{2}}{\theta_{2}{\left|h_{2}\right|}^{2}P_{1}+\sigma_{2}^{2}}\right)}{P_{s}+P_{c}}.$$

Thus, the EE optimization problem in direct-link mode can be summarized as

(23) $$\underset{P_{s}}{\max}\;EE_{k}^{direct}\;s.t.\;\;\;\;\;C_{1}:\;\Omega_{x_{1},U_{1,k}}^{direct}\geq \Omega_{x_{1},th}^{direct},\;\;\;\;\;\;\;\;\;\;C_{2}:\;\Omega_{x_{2},U_{2}}^{direct}\geq \Omega_{x_{2},th}^{direct},\;\;\;\;\;\;\;\;\;\;\;\;\;\;\;\;\;\;\;\;\;\;\;\;\;\;\;\;\;\;\;\;C_{3}:\;\theta_{1,k}{\left|h_{1,k}\right|}^{2}=\underset{i=1,\ldots ,K}{\max}\;\theta_{1,i}{\left|h_{1,i}\right|}^{2},\;C_{4}:\;P_{s}\geq P_{min}.$$

where $P_{c}$ is power consumed by the hardware circuit (mainly used for information decoding and re-encoding), and $P_{min}$ is the minimum power constraint to the BS. QoS requirements should be satisfied through these constraints to ensure normal communication.

### 4.2. EE Analysis in Cooperative Mode

The cooperative mode defined in this paper is used to add the $U_{1,k}-U_{2}$ communication link on the basis of direct-link mode, so the parameters considered in the EE optimization problem are more complicated.

The EE analytical expression in cooperative mode is given as

(24) $$EE_{k}^{coop}=\frac{\frac{T}{2}\left(R_{x_{1},U_{1,k}}^{coop}+R_{x_{2},U_{2}}^{coop}\right)}{\left(P_{s}+P_{c}\right)\frac{T}{2}+\left(P_{CR}-P_{EH}\right)\frac{T}{2}}\;=\frac{\frac{1}{2}\log_{2}\left(1+\frac{\left(1-\rho \right)\theta_{1,k}{\left|h_{1,k}\right|}^{2}P_{1}}{\sigma_{1,k}^{2}}\right)+\frac{1}{2}\log_{2}\left(1+\frac{\theta_{2}{\left|h_{2}\right|}^{2}P_{2}}{\theta_{2}{\left|h_{2}\right|}^{2}P_{1}+\sigma_{2}^{2}}+\frac{\theta_{3,k}{\left|h_{3,k}\right|}^{2}P_{r}}{\sigma_{3,k}^{2}}\right)}{\frac{1}{2}P_{s}+\frac{1}{2}P_{c}+\frac{1}{2}\eta \rho \theta_{1,k}{\left|h_{1,k}\right|}^{2}P_{s}-\frac{1}{2}\eta \rho \theta_{1,k}{\left|h_{1,k}\right|}^{2}P_{s}}$$

where $P_{EH}$ is the energy harvested by $U_{1,k}$ from the BS when acting as an EH with a power splitter, and $P_{CR}$ is the forwarding power of $U_{1}$ when transmitting information to $U_{2}$ as a cooperative relay. In this paper, we consider an ideal condition in which there is no additional energy loss in the process between energy harvesting and information forwarding in $U_{1,k}$, i.e., $P_{CR}=P_{EH}=\eta \rho \theta_{1,k}{\left|h_{1,k}\right|}^{2}P_{s}$.

It can be observed that EE in cooperative mode is not only determined by BS transmission power $P_{s}$, but it is also affected by the PS factor ρ of the SWIPT relay ($U_{1,k}$’s function in cooperative mode). The EE optimization problem in cooperative mode can be summarized as

(25) $$\underset{P_{s},\rho_{1,k}}{\max}\;EE_{k}^{coop}\;s.t.\;\;\;C_{5}:\;\Omega_{x_{1},U_{1}}^{direct}\geq \Omega_{x_{1},th}^{direct},\;\;\;\;\;\;\;\;\;C_{6}:\;\Omega_{x_{2},U_{2}}^{direct}<\Omega_{x_{2},th}^{direct},\;\;\;\;\;\;\;\;\;C_{7}:\;\Omega_{x_{1},U_{1,k}}^{coop}\geq \Omega_{x_{1},th}^{coop},\;\;\;\;\;\;\;\;C_{8}:\;\Omega_{x_{2},U_{2}}^{coop}\geq \Omega_{x_{2},th}^{coop},\;\;\;\;\;\;\;\;\;\;\;\;\;\;\;\;\;\;\;\;\;\;\;\;\;\;\;\;\;\;C_{9}:\;\theta_{1,k}{\left|h_{1,k}\right|}^{2}=\underset{i=1,\ldots ,K}{\max}\theta_{1,i}{\left|h_{1,i}\right|}^{2},\;\;C_{10}:\;P_{r}\geq P_{rmin},\;C_{11}:\;P_{s}\geq P_{min},\;C_{12}:\;0<\eta <1,\;C_{13}:\;0<\rho <1.$$

Compared with direct-link mode, the cooperative mode needs to optimize more parameters and consider more comprehensive QoS constraints.

For the dual-parameter optimization problem, the dual-layer solution can be performed, i.e., optimizing $P_{s}$ in the inner layer and ρ in the outer layer. However, at the same time, computational complexity will be increased.

If the optimal value of a single variable can be expressed by other variables through formula simplification and transformation, it can be directly replaced. In brief, it will be easier to solve the problem if dual-layer optimization can be transformed into single-layer optimization. For EE formulas of cooperative mode, the optimal relay PS factor ρ under fixed BS transmission power $P_{s}$ needs to be calculated and expressed by $P_{s}$; then, the single-layer iteration for the updated problem can be carried out to determine the maximum EE.

(26) $$EE_{k}^{coop}=\frac{\frac{T}{2}\left(R_{x_{1},U_{1,k}}^{coop}+R_{x_{2},U_{2}}^{coop}\right)}{\left(P_{s}+P_{c}\right)\frac{T}{2}+\left(P_{CR}-P_{EH}\right)\frac{T}{2}}\;=\frac{\log_{2}\left[\left(1+\frac{\left(1-\rho \right)\theta_{1,k}{\left|h_{1,k}\right|}^{2}P_{1}}{\sigma_{1,k}^{2}}\right)\left(1+\frac{\theta_{2}{\left|h_{2}\right|}^{2}P_{2}}{\theta_{2}{\left|h_{2}\right|}^{2}P_{1}+\sigma_{2}^{2}}+\frac{\theta_{3,k}{\left|h_{3,k}\right|}^{2}P_{r}}{\sigma_{3,k}^{2}}\right)\right]}{P_{s}+P_{c}}$$

The optimal EE under fixed $P_{s}$ can be deduced if $H\left(\rho \right)$ is optimal, which is expressed as

(27) $$H\left(\rho \right)=\left(1+\frac{\left(1-\rho \right)\theta_{1,k}{\left|h_{1,k}\right|}^{2}P_{1}}{\sigma_{1,k}^{2}}\right)\left(1+\frac{\theta_{2}{\left|h_{2}\right|}^{2}P_{2}}{\theta_{2}{\left|h_{2}\right|}^{2}P_{1}+\sigma_{2}^{2}}+\frac{\theta_{3,k}{\left|h_{3,k}\right|}^{2}P_{r}}{\sigma_{3,k}^{2}}\right)\;=A\rho^{2}+B\rho +C,$$

where

(28) $$A=-\frac{\theta_{1,k}^{2}\theta_{3,k}{\left|h_{1,k}\right|}^{4}{\left|h_{3,k}\right|}^{2}\eta P_{1}P_{s}}{\sigma_{1,k}^{2}\sigma_{3,k}^{2}},$$

(29) $$B=\left(1+\frac{\theta_{1,k}{\left|h_{1,k}\right|}^{2}P_{1}}{\sigma_{1,k}^{2}}\right)\frac{\theta_{1,k}\theta_{3,k}{\left|h_{1,k}\right|}^{2}{\left|h_{3,k}\right|}^{2}\eta P_{s}}{\sigma_{3,k}^{2}}-\left(1+\frac{\theta_{2}{\left|h_{2}\right|}^{2}P_{2}}{\theta_{2}{\left|h_{2}\right|}^{2}P_{1}+\sigma_{2}^{2}}\right)\frac{\theta_{1,k}{\left|h_{1,k}\right|}^{2}P_{1}}{\sigma_{1,k}^{2}},$$

(30) $$C=\left(1+\frac{\theta_{1,k}{\left|h_{1,k}\right|}^{2}P_{1}}{\sigma_{1,k}^{2}}\right)\left(1+\frac{\theta_{2}{\left|h_{2}\right|}^{2}P_{2}}{\theta_{2}{\left|h_{2}\right|}^{2}P_{1}+\sigma_{2}^{2}}\right).$$

Thus, the optimal PS factor $\rho^{*}$ and corresponding $H\left(\rho^{*}\right)$ can be written as

(31) $$\rho^{*}=-\frac{B}{2A},$$

(32) $$H_{opt}=H\left(\rho^{*}\right)=H^{*}{\left(P_{s}\right)}_{\;}$$

Through (31) and (32), $\underset{P_{s},\rho }{\max}\;EE_{k}^{coop}$ can be simplified into $\underset{P_{s}}{\max}\;EE_{k}^{coop}$.

## 5. Algorithm for EE Optimization

As analyzed in Section 4, EE optimization problems in the two different modes are both fractional programming problems. Thus, in this section, the *DinkelBach* iterative algorithm [34] is considered to maximize EE. Moreover, another algorithm based on the golden section [35] method, named GS-*DinkelBach*, is proposed to shorten the calculation steps in the *DinkelBach* algorithm and obtain lower computational complexity.

### 5.1. DinkelBach Iterative Algorithm

Taking EE optimization in cooperative mode as an example, the main influential element of the simplified EE optimization problem is the BS transmission power $P_{s}$, so it can be regarded as the BS transmission power optimization problem, which is a typical fractional programming problem. To solve such problems, the *DinkelBach* algorithm can be utilized.

Following from Section 4.2, through transformation, the EE formula can be expressed as

(33) $$EE_{k}^{coop}=\frac{\log_{2}H^{*}\left(P_{s}\right)}{P_{s}+P_{c}}.$$

Let the total achievable rate of the network $R\left(P_{s}\right)=\log_{2}H^{*}\left(P_{s}\right)$ and the total power consumption $E\left(P_{s}\right)=P_{s}+P_{c}$. For $R\left(P_{s}\right)\geq 0$ and $E\left(P_{s}\right)\geq 0$, the premise of achieving maximum energy efficiency $q^{*}$ is that the optimal BS transmission power and corresponding $R\left(P_{s}{}^{*}\right)$ and $E\left(P_{s}{}^{*}\right)$ are calculated and obtained, so the fractional problem can be transformed into a subtractive problem, i.e.,

(34) $$\underset{P_{s}}{\max}\;R\left(P_{s}\right)-q^{*}E\left(P_{s}\right)=R\left(P_{s}{}^{*}\right)-q^{*}E\left(P_{s}{}^{*}\right)=0.$$

Thus, the EE optimization problem is changed to

(35) $$\underset{P_{s}}{\max}\;\log_{2}H^{*}\left(P_{s}\right)-q\left(P_{s}+P_{c}\right).\;s.t.\;\;C_{5}-C_{13}.$$

The *DinkelBach* iterative algorithm for maximum EE in cooperative mode is implemented in Algorithm 1.

**Algorithm 1.***DinkelBach* iterative algorithm for EE optimization in cooperative mode.

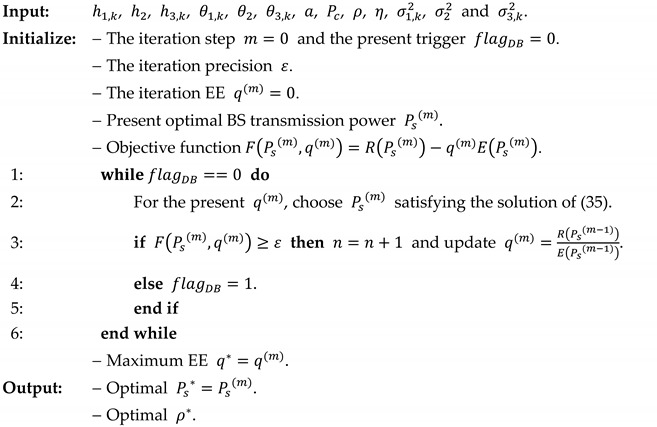



### 5.2. GS-DinkelBach Iterative Algorithm

In Algorithm 1, the essence of step 2 is still the traversal method, which should be optimized. For the objective function $F\left(P_{s},q\right)$ in (35), it can be proved that $F^{\prime \prime }\left(P_{s},q\right)<0$, so it is a concave function with unimodality. In this condition, a solution using the golden section could be employed to reduce the complexity of the searching optimal value. Using the golden section method, the probe point can be continuously updated by comparing the corresponding function value. As an algorithm similar to the traditional dichotomy, its complexity is much lower than that of the traversal scheme.

Let the feasible searching interval be $\left[\alpha_{n},\beta_{n}\right]$. If the function value at the probe point $\lambda_{n}$ is greater than that at the probe point $\mu_{n}$, the search interval will be updated as $\left[\alpha_{n+1},\beta_{n+1}\right]=\left[\alpha_{n},\mu_{n}\right]$. Otherwise, the search interval will be updated as $\left[\alpha_{n+1},\beta_{n+1}\right]=\left[\lambda_{n},\beta_{n}\right]$. The probe points satisfy

(36) $$\beta_{n}-\lambda_{n}=\mu_{n}-\alpha_{n},$$

(37) $$\beta_{n+1}-\alpha_{n+1}=\gamma \left(\beta_{n}-\alpha_{n}\right).$$

By combining (36) and (37), expressions of the probe points could be obtained,

(38) $$\lambda_{n}=\alpha_{n}+\left(1-\gamma \right)\left(\beta_{n}-\alpha_{n}\right),$$

(39) $$\mu_{n}=\alpha_{n}+\gamma \left(\beta_{n}-\alpha_{n}\right).$$

Assuming that $F\left(\lambda_{n}\right)\;>\;F\left(\mu_{n}\right)$, $\beta_{n+1}=\mu_{n}$ and

(40) $$\begin{array}{lll} \mu_{n+1} & = & \alpha_{n+1}+\gamma \left(\beta_{n+1}-\alpha_{n+1}\right)\\ & = & \alpha_{n}+\gamma \left(\mu_{n}-\alpha_{n}\right)\\ & = & \alpha_{n}+\gamma \left(\alpha_{n}+\gamma \left(\beta_{n}-\alpha_{n}\right)-\alpha_{n}\right)\\ & = & \alpha_{n}+\gamma^{2}\left(\beta_{n}-\alpha_{n}\right) \end{array}$$

Set $\gamma^{2}=1-\gamma$, i.e., $\gamma =\frac{\sqrt{5}+1}{2}\approx 0.618$; thus, there exists only one probe point that needs to be updated each time. In conclusion, formulas for updating the probe points based on the golden section are described as

(41) $$\lambda_{n}=\alpha_{n}+0.382\left(\beta_{n}-\alpha_{n}\right),$$

(42) $$\mu_{n}=\alpha_{n}+0.618\left(\beta_{n}-\alpha_{n}\right).$$

The implementation steps of the golden section algorithm for BS transmission power selection are presented in Algorithm 2.
**Algorithm 2.** Golden section algorithm for power selection.
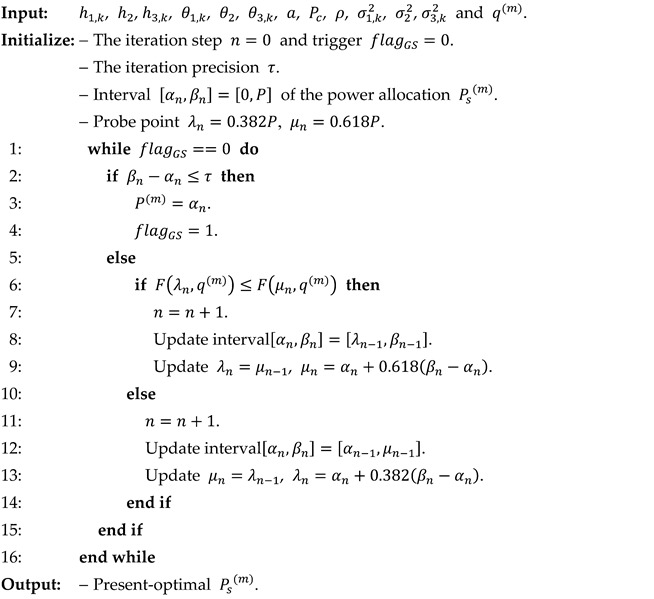


As the combination of the golden section and *DinkelBach*, the GS-*DinkelBach* iterative algorithm can not only reduce the complexity of the searching optimal value but also obtain the maximum EE.

## 6. Simulation Results

In this section, simulation and numerical analyses are conducted on the decoding and EE performance of different users in different states. Moreover, the superiority of the proposed NOMA–SWIPT cooperative relay system and GS-*DinkelBach* iterative algorithm is verified. The parameters of the simulations are shown in Table 1.

**Figure 3:**
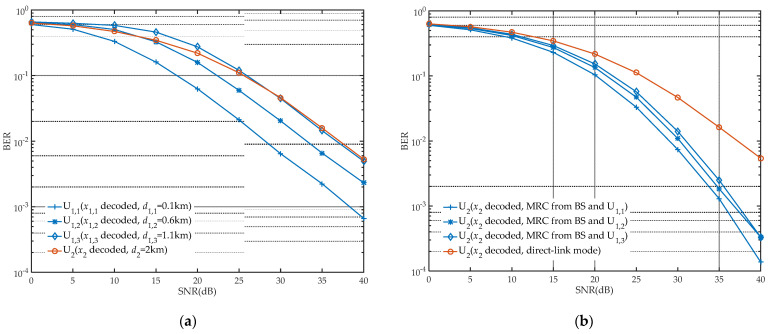
Decoding performance of users: (**a**) in direct-link mode ($P_{s}=1$, a=0.2); (**b**) in cooperative mode ($P_{s}=1$, a=0.2, ρ=0.6).

**Figure 4:**
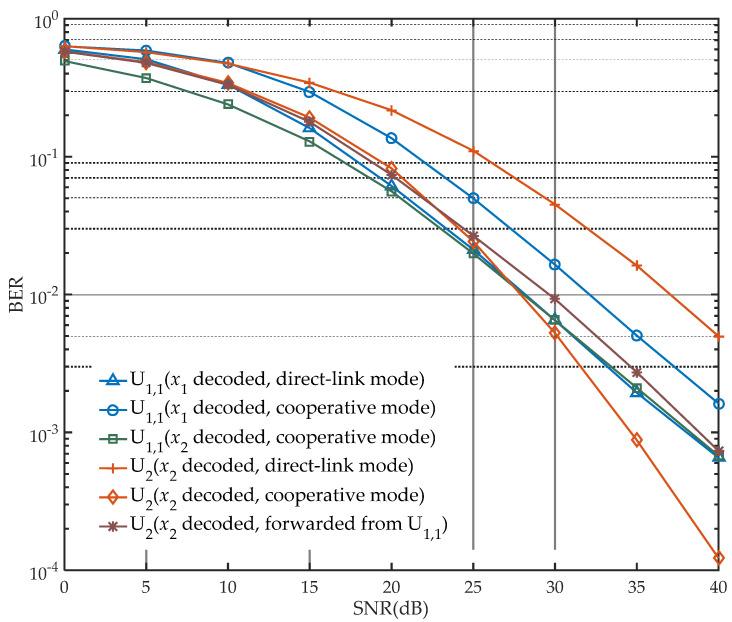
Decoding performance of users after user selection ($P_{s}=1$, a=0.2, ρ=0.6).

In order to study a relatively realistic network model, we simulated the process of channel transmission through Monte Carlo simulation. In these simulations, the distance between the BS and far user $U_{2}$ is fixed, and three near users $U_{1,k}$ (k=1,2,3) are randomly located (although the distances are fixed at 0.1 km, 0.6 km and 1.1 km, respectively, and the angles between near users and far user are unknown, so the two-dimensional topology model of the system is still random). Moreover, channel coefficients in communication links are also generated randomly using Raleigh fading. Through simulated channel transmission, the decoding performance of users in different modes is shown in Figure 3 and Figure 4.

Figure 3 shows the decoding performance of $U_{1,k}$ and $U_{2}$ in different modes. In Figure 3a, for near users, it can be seen that the BER is affected by the distances between users and the BS, which gradually increases with the lengthened distance and is even worse than that of $U_{2}$ in low SNR, referring to $U_{1,3}$. This is due to the utilization of SIC technology, where more energy is allocated for the signal $x_{2}$ required by $U_{2}$. To decode the required signal $x_{1}$, $U_{1,k}$ has to inevitably face the interference of residual $x_{2}$. Adding the distance influence, the decoding performance of $U_{1,3}$ is unsuitable to be a part of the proposed networks. From Figure 3b, it can be observed that $U_{2}$ could achieve higher performance gain in cooperative mode than in direct-link mode, where MRC is exploited in $U_{2}$ to obtain extra gain of $x_{2}$ from $U_{1,k}$. As analyzed in Section 3, the premise of ensuring forwarding quality is the higher performance of $\mathrm{BS}-U_{1,k}$ link communication. Considering the transmission requirements of communication distances, the $\mathrm{BS}-U_{1,1}-U_{2}$ link is the optimal performance communication link, which verifies the feasibility of the PRS scheme from the perspective of decoding performance.

Under the condition of user selection, Figure 4 summarizes the decoding performance of $U_{1,1}$ and $U_{2}$ in two modes. The performance of $U_{1,1}$ to decode $x_{1}$ in direct-link mode is similar to the performance of decoding $x_{2}$ in cooperative mode. This is due to the influence of residual $x_{2}$ when $x_{1}$ is decoded in direct-link mode and additional loss caused by power splitting of $U_{1,1}$ in cooperative mode. However, the decoding performance in these two cases is better than that of $U_{1,1}$ decoding $x_{1}$ in cooperative mode, which experiences power splitting and information decoding and is affected by losses of both the above. From the perspective of the transmission link, the BER performance of $U_{2}$ receiving $x_{2}$ through $U_{1,1}-U_{2}$ link transmission is also presented. Since only $x_{2}$ is transmitted, for a single link, the far user only decodes $x_{2}$, and there is no redundant interference, so its decoding performance can be an order of magnitude higher than $U_{2}$ decoding $x_{2}$ in direct-link mode under high SNR conditions. Through MRC, the performance of $U_{2}$ decoding $x_{2}$ is improved and is even better than that of others when the noise impact is sufficiently low. Overall, it should be noted that the decoding performance of the total network is not ideal because of the imperfect SIC (a difficult decision in decoding, which leads signal detection error) in actual simulation.

**Figure 5:**
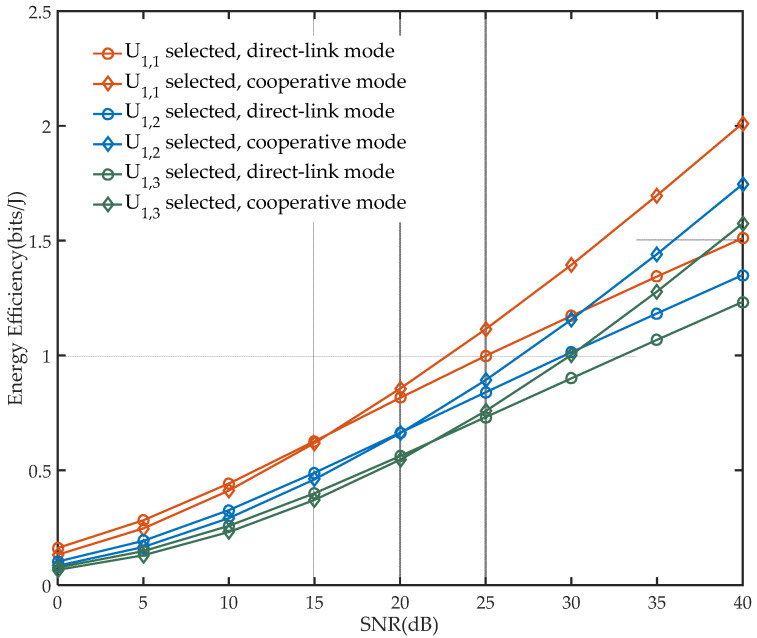
EE performance of networks under different user selections ($P_{s}=10$, a=0.2, ρ=0.6).

**Figure 6:**
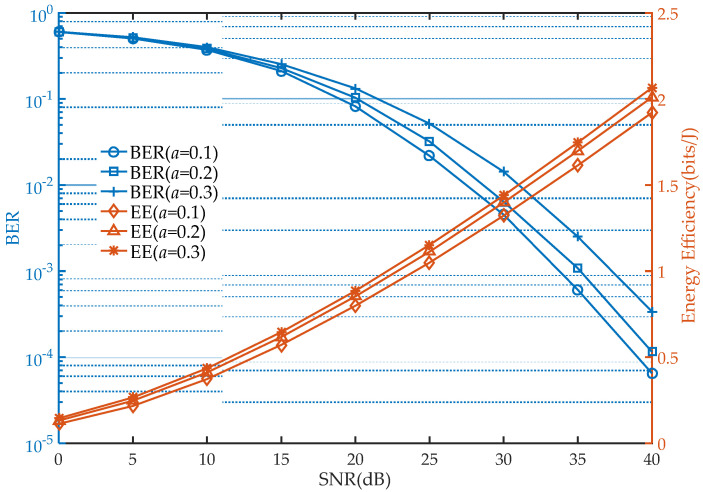
EE and BER performance comparison in cooperative mode under different BS power allocation factors ($P_{s}=10$, ρ=0.6).

To analyze the comprehensiveness performance of the system, the energy efficiency of the network under different modes and user selections is presented in Figure 5, and the BER and EE performance in the different BS power allocation factor is shown in Figure 6.

As shown in Figure 5, when the SNR is low, the EE of the network in direct-link mode is higher than that in cooperative mode. This is due to the fact that the achievable rate of $x_{2}$ transmitted from the cooperative relay under the condition of low SNR cannot make up for the achievable rate of $x_{1}$ after power splitting in the relay. Considering the fading led by the increased distance, $U_{1,1}$ should be selected for better EE performance, and the network could receive cooperative EE gain in a lower SNR compared with other selections.

It should be noted that when the BS power allocation is dynamic, the influence of EE and BER on network performance is inconsistent, which is verified in Figure 6. As the power allocation factor increases, the EE of the network in cooperative mode becomes higher but at the same time is accompanied by a decrease in BER in the far user. Although the influence of the BS power allocation factor on the network EE and BER is monotonous, considering the overall performance, there still exists a trade-off, and future work could focus on this.

**Figure 7:**
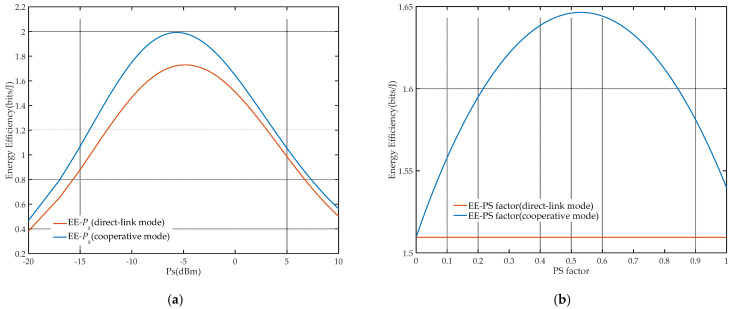
Influence curves of different parameters on EE: (**a**) BS transmission power $P_{s}$ (a=0.2, ρ=0.6); (**b**) relay PS factor ρ (a=0.2, $P_{s}=1$).

**Figure 8:**
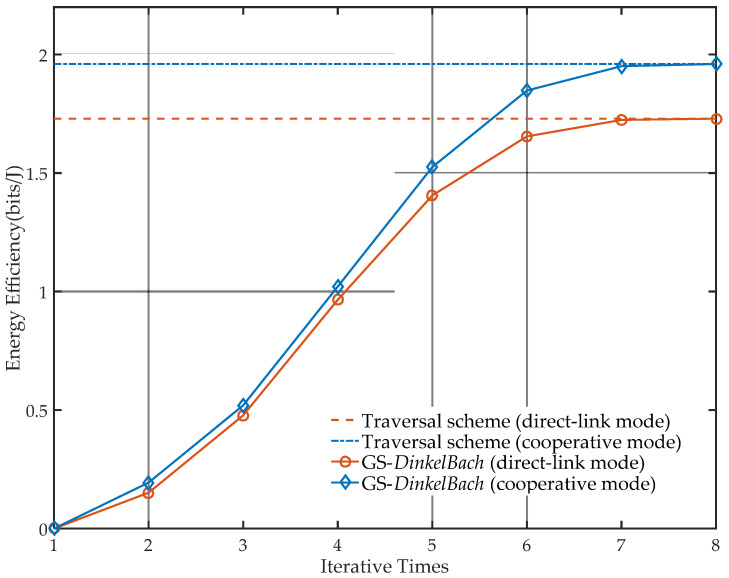
Network EE curves in different modes (a=0.2).

Figure 7 shows the influence curves of BS transmission power $P_{s}$ and the relay PS factor ρ on EE performance in the two modes. As shown in Figure 7a, on the premise of fixed ρ, the network EE will reach a peak value, which is the optimal EE with the corresponding optimal $P_{s}$. It can be clearly observed that as the fractional denominator of the EE formula, network energy consumption has a greater impact on network performance as with the continuous increase in $P_{s}$, so the curve will show a downward trend after reaching the peak value and gradually tend to 0. The overall curve presents a unimodal characteristic as analyzed in Section 5. On the other hand, as shown in Figure 7b, when $P_{s}$ is fixed, there also exists an optimal ρ to obtain the corresponding optimal EE in cooperative mode. It could be concluded that the network EE is a concave function on BS transmission power and the relay PS factor. Thus, the joint optimization of BS transmission power and the relay PS factor is meaningful, and the iterative algorithm could be utilized, as shown in Figure 8.

As can be seen in Figure 8, the GS-*DinkelBach* iterative algorithm and the traversal scheme are compared to prove the convergence of the proposed algorithm and the accuracy of the results. Through the algorithm, the EE reaches an optimal value through eight iterations in direct-link mode and cooperative mode. With the increase in iteration times, the EEs of the two different modes both gradually converge into stable values. Comparing the GS-*DinkelBach* algorithm with the traditional *DinkelBach* algorithm, the computational complexity of the proposed algorithm is $O\left(8\log_{1/0.618}\left(N\right)\right)$, while the computational complexity of the traditional *DinkelBach* algorithm is $O\left(8N\right)$. The algorithm proposed in this paper has lower computational complexity, and the superiority becomes more significant as the number of searching steps N of $P_{s}$ increases.

**Figure 9:**
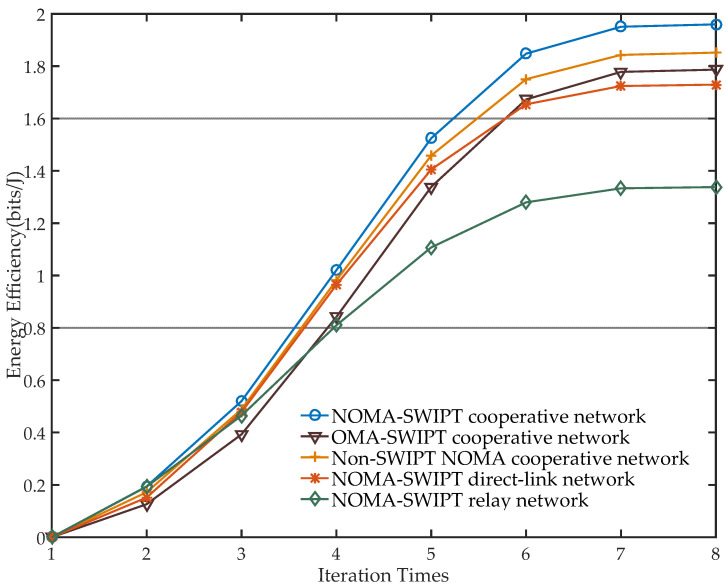
Comparison of EE performance in different system models (a=0.2).

The purpose of the simulation in Figure 9 is to compare the EE performance of different system models. It can be seen from Figure 9 that the NOMA–SWIPT cooperative relay network proposed in this paper has performance advantages, of which the EE composed of the same parameters is higher. As the iteration increases, the differences between EE performance in different models become more obvious.

Under the condition of fixed parameters, the power allocation scheme of NOMA technology has better network EE gain than the frequency division scheme of OMA because of its higher spectrum efficiency. When SWIPT is not taken into consideration, the relay no longer possesses the function of harvesting energy from the BS, which has to use extra power for signal forwarding, so the energy consumption of the system increases and the EE performance decreases. The EE performance of the network only using direct-link or cooperative communication is lower, because these two models do not receive and harvest as much information and energy as possible. The EE gain statistics obtained by the GS-*DinkelBach* algorithm are shown in Table 2.

As shown in Table 2, the optimal EE of the network architecture proposed in this paper is much better than that of the NOMA–SWIPT relay network. Moreover, compared with the conventional non-SWIPT–NOMA cooperative network and the OMA–SWIPT cooperative network, this architecture also has a certain EE gain. Figure 9 confirms the advantages of the integration of NOMA, SWIPT, and relay technology.

## 7. Conclusions

In this paper, a cooperative NOMA–SWIPT network is established, and a time-division resource allocation rule is proposed. Based on the network characteristics, the EE optimization problems in direct-link mode and cooperative mode are derived, and a GS-*DinkelBach* iterative algorithm is proposed and utilized to achieve the optimal EE. Simulation and numerical results present the decoding and EE performance of the network and verify that the algorithm proposed in this paper can obtain accurate and considerable EE gains with lower computational complexity. In addition, it is noted that the system model has better EE performance than that of traditional models, which confirms that the integration of NOMA, SWIPT, and relay technologies has a performance advantage. Therefore, NOMA–SWIPT cooperative relay networks have great potential for further exploration in future communications.

## Figures and Tables

**Table 1:** Parameters and corresponding values of simulations.

Parameters	Values
Modulation method	4QAM
Numbers of near users	K=3
Distance between BS and far user $U_{2}$	$d_{2}=2\;\mathrm{km}$
Propagation coefficients	ς=1,ϱ=3 [26]
Efficiency of energy harvester	η=0.7
Hardware circuit energy consumption	$P_{c}=0.05\;W$
Target data rates (bit per channel use)	$R_{1}=R_{2}=0.1$
Times of Monte Carlo simulation	100,000

**Table 2:** Optimal energy efficiency in different network models.

Network Models	Optimal EE (Bits/J)	Performance Improvement
NOMA–SWIPT cooperative network	1.9601	
OMA–SWIPT cooperative network	1.7870	0.40 dB
Non-SWIPT–NOMA cooperative network	1.8521	0.25 dB
NOMA–SWIPT direct-link network	1.7295	0.54 dB
NOMA–SWIPT relay network	1.3382	1.66 dB

## Data Availability

Not applicable.
